# Clot Mayhem: A Case of May-Thurner Syndrome

**DOI:** 10.7759/cureus.3899

**Published:** 2019-01-16

**Authors:** Asrar Ahmad, Muhammad A Zain, Ammar A Ashfaq, Zain Ali, Muhammad Arslan Cheema

**Affiliations:** 1 Internal Medicine, Abington Memorial Hospital, Abington, USA; 2 Internal Medicine, Sheikh Zayed Medical College and Hospital, Rahim Yar Khan, PAK

**Keywords:** may thurner syndrome, iliac vein compression syndrome, cockett's syndrome, deep venous thrombosis, dvt, recurrent dvt, warfarin failure, iliofemoral venous stenting, ir guided thrombectomy

## Abstract

May-Thurner syndrome (MTS), also known as the iliac vein compression syndrome, is an anatomic anomaly in which the compression of the left common iliac vein by an overlying right common iliac artery leads to blood stasis, which predisposes to deep venous thrombosis (DVT) in the iliofemoral venous system. We present a case of a female with a history of DVT and currently on warfarin with a therapeutic international normalized ratio (INR), admitted with left leg swelling, redness, and intractable pain. Venous Doppler scan confirmed a massive DVT from the level of the left common femoral vein to the popliteal vein. The clot recurred after an unsuccessful trial of argatroban, in-line pharmacokinetic thrombolysis with local catheter-based alteplase infusion, and interventional radiology (IR)-guided mechanical thrombectomy. Subsequently, the patient was diagnosed as MTS with overlying left common iliac vein compression, as evident on venous Doppler ultrasound. She was managed successfully by venous stent placement and ongoing systemic anticoagulation with fondaparinux.

## Introduction

May-Thurner syndrome (MTS) is a rare cause of iliofemoral venous outflow impedance secondary to extrinsic venous compression in the iliocaval venous territory. With partial venous obstruction, the condition can be asymptomatic or associated with chronic ipsilateral leg swelling, but venous thrombosis can occur with critical venous outflow obstruction. MTS must be kept as a substantial differential in evaluating a young female patient with persistent unilateral leg swelling and/or deep venous thrombosis (DVT) of no apparent cause [1-3]. Medical treatment with heparin or warfarin is associated with the suboptimal control of DVT as well as a substantial financial burden on the healthcare system [4]. Therefore, an aggressive approach is mandatory to prevent and treat extensive recurrent DVT. Endovascular treatment with or without venous stenting usually resolves DVT in the presence of ongoing anticoagulation but in rare cases, extensive recurrent DVT can occur after the venous stenting, which may require venous bypass surgery. However, we present a case of MTS with extensive recurrent DVT, which was managed successfully with venous stenting. The purpose of our case is to help make the physician aware of warfarin failure associated with a rare cause of extensive DVT and to add to a growing body of literature in which venous stenting has been used to treat MTS successfully.

## Case presentation

A 23-year-old Caucasian female with a past medical history of heparin-induced thrombocytopenia (HIT), deep venous thrombosis (DVT) in her left lower extremity (LLE), and pulmonary embolism (PE) came to the hospital with low-grade fever, worsening LLE swelling, and redness for two weeks. She denied any recent history of trauma, prolonged immobility, chest pain, shortness of breath, or weight loss. She also denied any history of alcoholism, tobacco, or illicit drug use. Her family history was significant for systemic lupus erythematosus (SLE) in her mother and factor V Leiden mutation in her father. Her medication included coumadin and over-the-counter painkillers. Her initial vitals revealed a low-grade fever of 100.6 F. The physical exam showed LLE swelling extending up to the proximal calf, with mild diffuse redness of the skin and no demarcation. Her lungs were clear to auscultation bilaterally, and her oxygen saturation was 98% on room air.

Investigation

**Figure 1:**
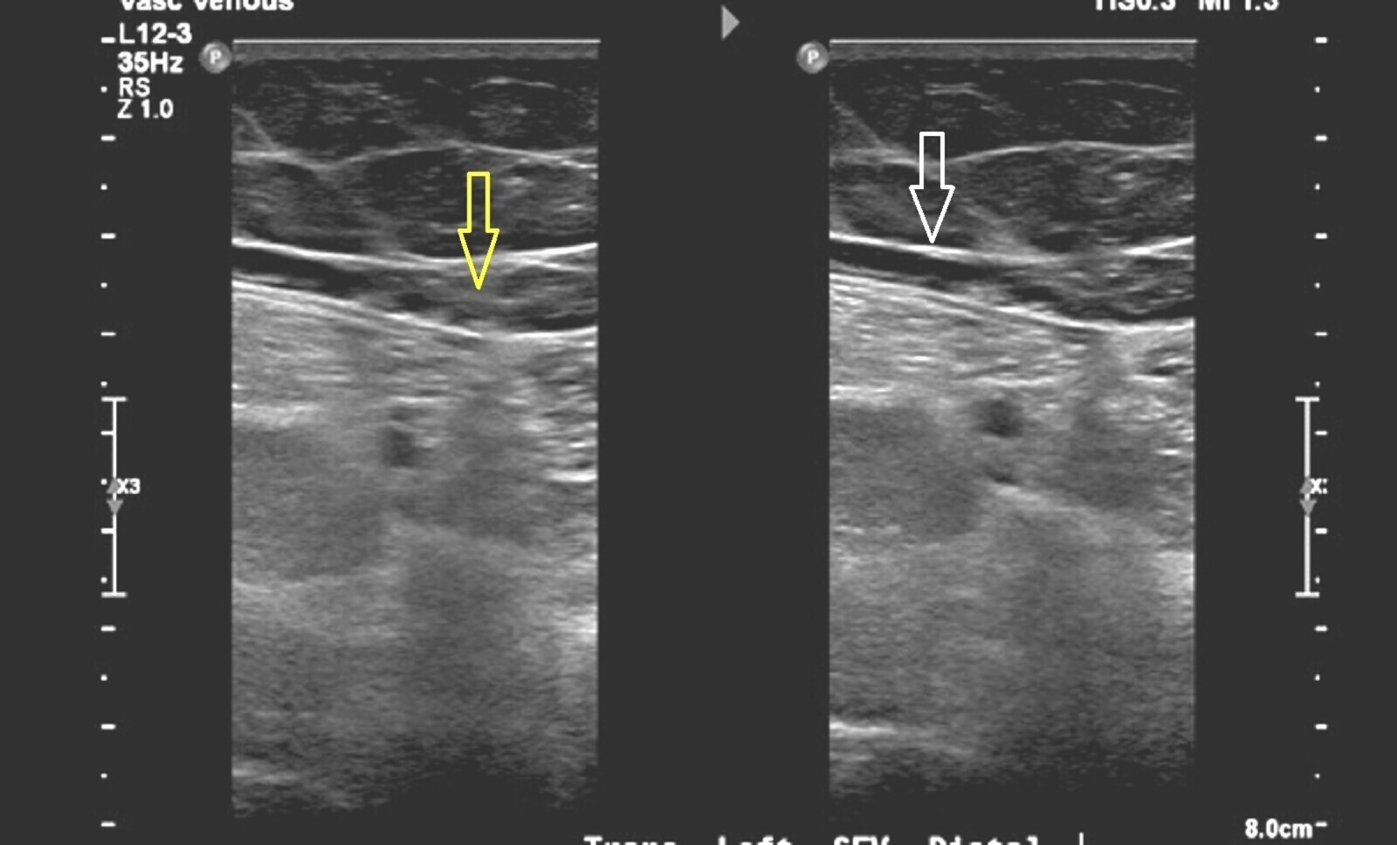
Showing LCFV compression (white arrow) with large intraluminal thrombus (yellow arrow) distal to compression LCFV: Left common femoral vein

Her blood cultures were obtained and admitted to the general medical floor on broadspectrum antibiotics and pain medications. On the first day of admission, her blood workup was significant for the following: international normalized ratio (INR) = 2.7; partial thromboplastin time (PTT) = 45 seconds; blood urea nitrogen (BUN) = 13 milligram per deciliter (mg/dL); and serum creatinine = 0.74 mg/dL. Complete blood count was evident for white blood cell (WBC) count = 5.5 k/UL; hemoglobin = 10.4 g/dL; and platelet count = 324 k/UL. Urinalysis and chest X-ray (posteroanterior (PA) view) were within standard limits. Venous Doppler of her LLE was significant for a large DVT in the left common femoral vein. Ultrasound also revealed an abnormal compression of her left common femoral (Figure 1) and popliteal vein.

She was started on argatroban infusion due to her history of HIT. She was initially started on broadspectrum antibiotics, which were stopped eventually upon negative culture data and no source of infection.

Differential diagnosis

Upon presentation, we suspected either LLE acute cellulitis, recurrent DVT, hypercoagulability or warfarin failure due to skipped doses, or thrombophilia associated with antiphospholipid antibody syndrome (APLAS). But our suspicion of MTS grew after getting a normal blood culture, WBC count, therapeutic range INR upon presentation, and normal blood titers for the anticardiolipin antibody, antinuclear antibody, and factor V Leiden.

Treatment

Due to the extent of the thrombosis and persistent severe pain, it was decided to send her for IR-guided mechanical thrombectomy. She underwent successful in-line pharmacokinetic thrombolysis with alteplase and IR-guided mechanical thrombectomy. The patient remained on argatroban infusion for four days. Her LLE venous Doppler scan was repeated, which revealed a recurrence of the clot. Vascular surgery was taken on board, and she underwent iliofemoral venous stent placement. Eventually, she was discharged from the hospital on Day 10 on fondaparinux.

Outcome and follow-up

The patient followed up with the hematologist after four weeks of her discharge. She had a repeat venous Doppler of her left leg, which showed no recurrence of her DVT.

## Discussion

Although McMurrich et al. first described the association of left lower extremity swelling with left iliac vein compression in 1908, the full description of MTS was not available until 1957, when May and Thurner discovered the anatomical variant, an overriding right iliac artery compressing the left common iliac vein against the lumbar spine, during autopsies of 22% of cadavers [4]. In 2004, Kibbe et al. reported a similar prevalence of this anatomical variant in a retrospective study [5]. MTS is relatively common in young females. The most common anatomical variation of MTS is due to the compression of the left iliac vein between the overlying right common iliac artery and the fifth lumbar vertebrae, but variants originating from the right side have also been reported rarely. Acquired MTS has also been reported in patients with scoliosis [4].

Two theories have been postulated for MTS-associated venous outflow obstruction. First, it is believed that chronic pulsations of the right iliac artery induce intimal hyperplasia of the underlying left common iliac vein, thus causing venous luminal shrinkage. Second, it is also believed that physical entrapment of the left common iliac vein by the overlying right iliac artery causes the external compression [1].

The clinical presentation of MTS depends upon the degree of venous outflow obstruction. In patients with partial or under 70% obstruction, luminal stenosis may remain asymptomatic or may present with chronic leg swelling, nocturnal leg size discrepancy, venous claudication, leg skin pigmentation, superficial thrombophlebitis, or varicosities of legs [4]. A complete, or over 70%, luminal occlusion may present with acute onset lower extremity swelling, pain, or DVT [3]. The risk of DVT in MTS is increased manyfold if the congenital or acquired hypercoagulation is present simultaneously. A comprehensive hypercoagulability blood workup should be considered before MTS consideration. This includes investigating the levels of prothrombin, antithrombin, protein C, protein S, and factor V Leiden mutation or a history of oral contraceptive pills intake [4,6].

Despite the high prevalence of anatomical variants, about 22%, MTS has been reported in only 2%-3 % of cases of lower extremity DVT patients. Such a low occurrence of clinically symptomatic MTS can be explained by missed diagnoses, which is supported by the fact that 60% of all DVTs are left-sided and the most common anatomical variant of MTS is also left-sided in origin [5]. So the exploration of the underlying pathology for DVT may be halted once other common risk factors have been identified early on, such as pregnancy, oral contraceptive pill intake, or prolonged immobilization. Failure to correct this anatomical variation could act as a substrate for recurrent DVT and other complications, such as chronic ipsilateral leg swelling, pulmonary embolism, or iliac vein rupture. MTS has been reported in 28% of cases of iliac vein rupture [5]. Therefore, whenever a young female patient presents with acute or chronic lower limb swelling or symptoms of DVT, it is very crucial to investigate MTS to avoid serious complications.

MTS is diagnosed with radiological evidence of iliocaval venous compression. Since the anatomical defects associated with MTS are located high in the pelvic region, which is not well-visualized by Doppler ultrasonography (USG), Doppler USG holds very little sensitivity to diagnose the anatomical defects associated with MTS [5,7]. However, in our case, venous compression was evident with an LLE Doppler scan. Meanwhile, Doppler USG can easily detect DVT associated with MTS. A computed tomography (CT) scan of the abdomen and pelvis, contrast venography, CT angiography, magnetic resonance venography (MRV), or intravascular USG are preferred over Doppler USG for the better visualization of anatomical defects and degree of iliac vein compression [5,7]. The gold standard modality to diagnose MTS is conventional venography. Despite the availability of a wide range of diagnostic tools, no single test is accepted as diagnostic criteria. Even the same imaging test done at a different time in the same patient can provide different results because of different confounding factors such as the volume status of the patient. Therefore, a combination of tests should be used to precisely diagnose the condition [8]. These noninvasive imaging modalities may aid in correct venous catheterization for thrombus removal as well [5].

Treatment

Patients with MTS commonly remain asymptomatic and go unrecognized until symptoms develop. It is believed that long-term systemic anticoagulation alone, while indicated, is not sufficient to prevent long-term sequelae in MTS patients such as recurrent DVT and iliac vein rupture [4]. Our case was similar, as the patient developed recurrent DVT even on warfarin with therapeutic INR. So, a more invasive therapeutic strategy aims at better long-term results in symptomatic patients. Therefore, endovascular thrombolysis followed by venous stenting is considered the first line of treatment for symptomatic MTS with superimposed DVT [4]. The first and foremost issue to deal with is active iliofemoral venous thrombosis, which should be dealt with as early as possible to prevent impending lethal complications such as post-thrombotic syndrome, iliac vein rupture, and pulmonary embolism. The correction of an anatomical defect of MTS should be done after initial thrombolysis has been achieved [1,4].

Thrombolysis can be achieved through mechanical and pharmacological interventions or a combination of both for satisfactory outcomes. Traditionally, surgical thrombectomy alone, without venous stenting, was used to clear the thrombus, but it was associated with venous re-occlusion in 70% of cases, with iliofemoral thrombosis in the setting of iliocaval venous occlusion [7]. Therefore, a combination of percutaneous mechanical thrombectomy (PMT) and local catheter-based pharmacological thrombolysis is recommended nowadays. PMT reduces the clot burden as well as the local thrombolytic infusion time. Catheter-based pharmacological thrombolysis is achieved with a local infusion of urokinase or tissue plasminogen activator (t-PA) through the tip of a catheter locally at the site of thrombus [7]. In our case, in-line local pharmacological thrombolysis was achieved with the help of alteplase infusion through the catheter tip. It is recommended that local thrombolytic infusion should be continued for an additional 24-48 hours following clot lysis [5]. Local catheter-based thrombolysis is preferred over systemic thrombolytics because there is a high risk of major bleeding. Thrombolytic therapy is commonly performed to decrease the risk of major bleeding that can be seen with the use of systemic thrombolytic therapy. In patients with a higher clot burden, Moudgill et al. also suggested the placement of an inferior vena cava filter before any lower extremity intervention to prevent distal thromboembolism during lytic therapy [7].

When it comes to the correction of the anatomical defects of MTS, the treatment has evolved over the decades, ranging from conventional open surgical repair techniques (such as the creation of tissue slings to lift the iliac artery off the iliac vein, retro positioning of the overriding iliac artery, and venovenous bypass surgery) to less invasive endovascular repair techniques such as venous stenting [7]. Berger et al. was the first person who did successful iliac vein stenting in an MTS patient in 1995 [7]. After that, successful attempts at the correction of an anatomical defect of MTS using angioplasty and stenting dates back to 1998 when Binkert et al. reported 100% patency of the left iliac vein stents in eight cases [1]. However, Moudgill et al. reported a one-year patency rate of 95% in a study involving 113 patients of MTS with left iliac vein thrombosis who underwent catheter-based thrombolysis and iliac vein stenting [1]. Data has proven that two-year iliac vein stent patency rates are between 95% and 100%. Suwanabol et al. recommend the placement of large, self-expanding stents, about 12-14 mm in size, across the whole left iliac vein area, which is compressed as well as spanning into the inferior vena cava, to prevent accidental stent migration [5].

Following stent deployment, it is recommended to get repeat imaging of iliac veins to document proper stent placement. Endovascular stenting has been associated with several complications such as retroperitoneal hemorrhage, early in-stent thrombosis, stent migration, and stent fracture. Despite anticoagulation, several factors increase the risk of early in-stent restenosis, which includes recent trauma, thrombotic disease, thrombophilia, and stenting below the inguinal ligament. After endovascular thrombolysis and stent placement, patients are routinely recommended to take systemic anticoagulation for about six months to maintain venous patency and prevent in-stent restenosis [1,4-5,7]. In our case, the patient underwent thrombolysis and stent placement. Finally, long-term anticoagulation therapy for six months was administered to maintain venous patency and prevent in-stent restenosis. Therefore, a multidisciplinary approach, as indicated in our case, is important to manage this condition effectively.

## Conclusions

Our case highlights the need for MTS to be kept as a differential diagnosis in a young female patient who primarily presents with lower limb DVT because the uncorrected anatomical defects of MTS can lead to recurrent DVT, pulmonary embolism, or iliac vein rupture. Our case also highlights the successful usage of iliofemoral venous stenting after a failed attempt at in-line pharmacokinetic and IR-guided mechanical thrombolysis alone. Systemic anticoagulation alone is insufficient for managing MTS cases, but it is generally recommended as routine treatment for DVT. The exact duration of systemic anticoagulation is debatable, but it is highly recommended that anticoagulation is administered for a six-month period to maintain the patency of the vein.
